# 2-Hy­droxy-5-nitro-*N*-phenyl­benzamide

**DOI:** 10.1107/S1600536810024621

**Published:** 2010-06-26

**Authors:** Abdul Rauf Raza, Bushra Nisar, M. Nawaz Tahir

**Affiliations:** a Department of Chemistry, University of Sargodha, Sargodha, Pakistan; b Department of Physics, University of Sargodha, Sargodha, Pakistan

## Abstract

The mol­ecule of the title compound, C_13_H_10_N_2_O_4_, is  almost planar with a dihedral angle between the benzene rings of 1.99 (13)°. The nitro group and its parent benzene ring are oriented at a dihedral angle of 7.6 (3)°. Intra­molecular C—H⋯O and N—H⋯O hydrogen bonds form two planar *S*(6) motifs. Inter­molecular O—H⋯O=C hydrogen bonds join mol­ecules into chains extending along the *c* axis.

## Related literature

For similar structures, see: Raza *et al.* (2009*a* ▶,*b* ▶). For graph-set notation of hydrogen-bond motifs, see: Bernstein *et al.* (1995 ▶).
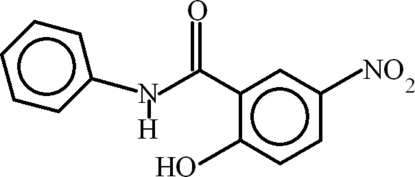


## Experimental

### 

#### Crystal data

C_13_H_10_N_2_O_4_
*M* *_r_* = 258.23
Monoclinic, 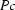

*a* = 9.9012 (2) Å
*b* = 4.7821 (1) Å
*c* = 12.3369 (4) Å
β = 97.919 (1)°
*V* = 578.56 (3) Å^3^
*Z* = 2
Mo *K*α radiation
μ = 0.11 mm^−1^
*T* = 296 K
0.34 × 0.12 × 0.10 mm

#### Data collection

Bruker Kappa APEXII CCD diffractometer
Absorption correction: multi-scan (*SADABS*; Bruker, 2009 ▶) *T*_min_ = 0.979, *T*_max_ = 0.988
4381 measured reflections
1042 independent reflections
966 reflections with *I* > 2σ(*I*)
*R*_int_ = 0.022

#### Refinement

*R*[*F* ^2^ > 2σ(*F* ^2^)] = 0.029
*wR*(*F* ^2^) = 0.071
*S* = 1.06
1042 reflections
173 parameters
2 restraints
H-atom parameters constrained
Δρ_max_ = 0.13 e Å^−3^
Δρ_min_ = −0.13 e Å^−3^

### 

Data collection: *APEX2* (Bruker, 2009 ▶); cell refinement: *SAINT* (Bruker, 2009 ▶); data reduction: *SAINT*; program(s) used to solve structure: *SHELXS97* (Sheldrick, 2008 ▶); program(s) used to refine structure: *SHELXL97* (Sheldrick, 2008 ▶); molecular graphics: *ORTEP-3 for Windows* (Farrugia, 1997 ▶) and *PLATON* (Spek, 2009 ▶); software used to prepare material for publication: *WinGX* (Farrugia, 1999 ▶) and *PLATON*.

## Supplementary Material

Crystal structure: contains datablocks global, I. DOI: 10.1107/S1600536810024621/gk2288sup1.cif

Structure factors: contains datablocks I. DOI: 10.1107/S1600536810024621/gk2288Isup2.hkl

Additional supplementary materials:  crystallographic information; 3D view; checkCIF report

## Figures and Tables

**Table 1:** Hydrogen-bond geometry (Å, °)

*D*—H⋯*A*	*D*—H	H⋯*A*	*D*⋯*A*	*D*—H⋯*A*
O1—H1⋯O4^i^	0.82	1.79	2.609 (2)	176
N2—H2*A*⋯O1	0.86	1.95	2.675 (2)	141
C2—H2⋯O4^i^	0.93	2.54	3.212 (3)	130
C9—H9⋯O4	0.93	2.26	2.853 (3)	121
C11—H11⋯O2^ii^	0.93	2.59	3.335 (4)	137

Symmetry codes: (i) 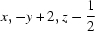; (ii) 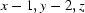.

**Fig. 1.**
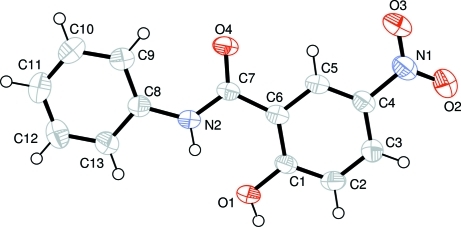
View of the title compound with the atom numbering scheme. The displacement ellipsoids are drawn at the 50% probability level. H-atoms are shown by small circles of arbitrary radii.

**Fig. 2.**
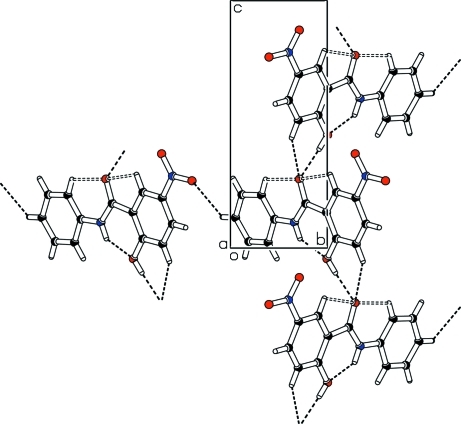
The partial packing (PLATON; Spek, 2009) which shows that molecules form polymeric chains extending along the c-axis. Hydrogen bonds are shown by dashed lines.

## supplementary crystallographic information

### Comment

The title compound (I), (Fig. 1) has been synthesized as a precursor for benzoxazepines.

Previously, we have reported the crystal structures of N-phenyl-2-hydroxy-3-nitrobenzamide (Raza *et al.*, 2009a). The title compound differs from it due to the attachement of nitro group at position-5 instead of position-3. We, also have reported the crystal structure of 2-hydroxy-5-nitrobenzamide (Raza *et al.*, 2009b) which is related to (I).

In (I), the phenyl rings, A (C1–C6) of 2-hydroxy-5-nitrobenzamide and B (C8–C13) attached with 2-hydroxy-5-nitrobenzamide are planar with r. m. s. deviation of 0.0027 Å and 0.0031 Å, respectively. The O-atom of hydroxy group is at a distance of 0.014 (3) Å from the mean square plane of parent ring A. Nitro group C (O2/N1/O3) is of course planar. The dihedral angle between A/B, A/C and B/C is 1.99 (13)°, 7.63 (33)° and 6.20 (34)°, respectively. There exist a weak intramolecular H-bonding of C—H···O type forming an S(5) and a S(6) ring motif (Bernstein *et al.*, 1995), whereas H-bonding of N—H···O type complete an S(6) ring motif. The intermolecular H-bonding of C—H···O and O—H···O types complete R_2_^1^(6) ring motif (Table 1, Fig. 2). The molecules are essentially stabilized in the form of one dimensional chains extending along the c-axis. However, weak interactions of C—H···O type form 2-dimensional polymeric sheets (Fig. 2).

### Experimental

A solution of *N*-pheny-2-hydroxybenzamide (5.3 g, 0.025 mol) in ethyl acetate (EtOAc) (25 mL) was added dropwise to a nitrating mixture of HNO_3_ (2.25 mL, 3.15 g , 0.05 mol) and H_2_SO_4_ (1.33 mL, 2.45 g, 0.025 mol) with constant stirring while the temperature was kept below 278 K. The reaction mixture was refluxed for 5 h, cooled to room temperature, neutralized with aqueous NaHCO_3_ (10%) and extracted with EtOAc (3 × 25 mL). The organic extract was combined, dried over anhydrous Na_2_SO_4_, filtered and concentrated under reduced pressure to afford reddish brown solid. The column chromatographic purification with 0, 2.5, and 5 % EtOAc in n-hexane (0.5 L each) over a silica gel packed column (25.5 cm) afforded the title compound I in 5th-34th fraction of 50 mL each upon leaving at room temperature.

### Refinement

In the absence of significant anomalous scattering effects, all Friedal pairs were merged. All H atoms were found in difference Fourier maps however for the refinement they were positioned geometrically with O–H = 0.82, N–H= 0.86 and C–H = 0.93 Å and refined as riding with *U*_iso_(H) = 1.2*U*_eq_(C, N) and  *U*_iso_(H) = 1.5*U*_eq_(O).

### Crystal data

**Table:**

C_13_H_10_N_2_O_4_	*F*(000) = 268
*M**_r_* = 258.23	*D*_x_ = 1.482 Mg m^−^^3^
Monoclinic, *P**c*	Mo *K*α radiation, λ = 0.71073 Å
Hall symbol: P -2yc	Cell parameters from 931 reflections
*a* = 9.9012 (2) Å	θ = 2.8–26.0°
*b* = 4.7821 (1) Å	µ = 0.11 mm^−^^1^
*c* = 12.3369 (4) Å	*T* = 296 K
β = 97.919 (1)°	Needle, colorless
*V* = 578.56 (3) Å^3^	0.34 × 0.12 × 0.10 mm
*Z* = 2	

### Data collection

**Table:**

Bruker Kappa APEXII CCD diffractometer	1042 independent reflections
Radiation source: fine-focus sealed tube	966 reflections with *I* > 2σ(*I*)
graphite	*R*_int_ = 0.022
Detector resolution: 8.20 pixels mm^-1^	θ_max_ = 25.3°, θ_min_ = 3.7°
ω scans	*h* = −11→11
Absorption correction: multi-scan (*SADABS*; Bruker, 2009)	*k* = −5→5
*T*_min_ = 0.979, *T*_max_ = 0.988	*l* = −14→14
4381 measured reflections	

### Refinement

**Table:**

Refinement on *F*^2^	Primary atom site location: structure-invariant direct methods
Least-squares matrix: full	Secondary atom site location: difference Fourier map
*R*[*F*^2^ > 2σ(*F*^2^)] = 0.029	Hydrogen site location: inferred from neighbouring sites
*wR*(*F*^2^) = 0.071	H-atom parameters constrained
*S* = 1.06	*w* = 1/[σ^2^(*F*_o_^2^) + (0.0447*P*)^2^ + 0.0284*P*] where *P* = (*F*_o_^2^ + 2*F*_c_^2^)/3
1042 reflections	(Δ/σ)_max_ < 0.001
173 parameters	Δρ_max_ = 0.13 e Å^−^^3^
2 restraints	Δρ_min_ = −0.13 e Å^−^^3^

### Special details

**Table:**

Geometry. Bond distances, angles etc. have been calculated using the rounded fractional coordinates. All su's are estimated from the variances of the (full) variance-covariance matrix. The cell esds are taken into account in the estimation of distances, angles and torsion angles
Refinement. Refinement of *F*^2^ against ALL reflections. The weighted *R*-factor *wR* and goodness of fit *S* are based on *F*^2^, conventional *R*-factors *R* are based on *F*, with *F* set to zero for negative *F*^2^. The threshold expression of *F*^2^ > σ(*F*^2^) is used only for calculating *R*-factors(gt) *etc*. and is not relevant to the choice of reflections for refinement. *R*-factors based on *F*^2^ are statistically about twice as large as those based on *F*, and *R*- factors based on ALL data will be even larger.

### Fractional atomic coordinates and isotropic or equivalent isotropic displacement parameters (Å^2^)

**Table:**

	*x*	*y*	*z*	*U*_iso_*/*U*_eq_	
O1	0.87642 (17)	0.9971 (4)	−0.04316 (13)	0.0503 (6)	
O2	1.3304 (2)	1.6021 (5)	0.27212 (18)	0.0782 (8)	
O3	1.2680 (2)	1.2889 (5)	0.38093 (16)	0.0688 (8)	
O4	0.88497 (17)	0.7133 (3)	0.27868 (13)	0.0484 (5)	
N1	1.25998 (19)	1.4015 (5)	0.29149 (17)	0.0502 (7)	
N2	0.78477 (17)	0.6615 (4)	0.10417 (14)	0.0398 (6)	
C1	0.9697 (2)	1.0963 (5)	0.03785 (17)	0.0374 (7)	
C2	1.0633 (2)	1.2998 (5)	0.01674 (19)	0.0452 (8)	
C3	1.1591 (2)	1.4007 (5)	0.09772 (19)	0.0440 (8)	
C4	1.1606 (2)	1.2957 (5)	0.20283 (19)	0.0395 (7)	
C5	1.0696 (2)	1.0964 (5)	0.22608 (17)	0.0382 (7)	
C6	0.9712 (2)	0.9923 (4)	0.14475 (17)	0.0353 (7)	
C7	0.8756 (2)	0.7790 (4)	0.18042 (17)	0.0356 (6)	
C8	0.6844 (2)	0.4564 (4)	0.11679 (19)	0.0378 (7)	
C9	0.6723 (3)	0.3201 (5)	0.2141 (2)	0.0467 (8)	
C10	0.5716 (3)	0.1191 (5)	0.2159 (2)	0.0562 (9)	
C11	0.4843 (3)	0.0530 (5)	0.1231 (3)	0.0565 (9)	
C12	0.4959 (2)	0.1904 (5)	0.0268 (2)	0.0563 (9)	
C13	0.5957 (3)	0.3893 (5)	0.0230 (2)	0.0494 (8)	
H1	0.88228	1.08314	−0.09982	0.0754*	
H2	1.06061	1.36856	−0.05404	0.0542*	
H2A	0.78731	0.71755	0.03824	0.0477*	
H3	1.22149	1.53593	0.08284	0.0528*	
H5	1.07351	1.02973	0.29727	0.0459*	
H9	0.73090	0.36301	0.27744	0.0561*	
H10	0.56322	0.02735	0.28111	0.0674*	
H11	0.41778	−0.08347	0.12537	0.0677*	
H12	0.43598	0.14877	−0.03597	0.0675*	
H13	0.60379	0.47916	−0.04261	0.0592*	

### Atomic displacement parameters (Å^2^)

**Table:**

	*U*^11^	*U*^22^	*U*^33^	*U*^12^	*U*^13^	*U*^23^
O1	0.0561 (9)	0.0674 (11)	0.0251 (9)	−0.0152 (8)	−0.0027 (7)	0.0045 (8)
O2	0.0822 (13)	0.0874 (15)	0.0612 (13)	−0.0397 (13)	−0.0041 (10)	−0.0068 (11)
O3	0.0754 (13)	0.0863 (14)	0.0391 (12)	−0.0147 (11)	−0.0117 (9)	0.0010 (10)
O4	0.0645 (10)	0.0525 (9)	0.0267 (9)	−0.0075 (8)	0.0014 (7)	0.0006 (7)
N1	0.0480 (11)	0.0581 (13)	0.0424 (13)	−0.0011 (10)	−0.0009 (9)	−0.0086 (10)
N2	0.0470 (11)	0.0462 (11)	0.0258 (10)	−0.0058 (8)	0.0041 (8)	0.0007 (8)
C1	0.0381 (11)	0.0458 (12)	0.0275 (12)	0.0011 (10)	0.0016 (8)	−0.0024 (9)
C2	0.0503 (14)	0.0554 (14)	0.0301 (13)	−0.0010 (11)	0.0060 (10)	0.0036 (10)
C3	0.0416 (12)	0.0488 (14)	0.0419 (14)	−0.0062 (10)	0.0067 (10)	−0.0030 (11)
C4	0.0372 (11)	0.0447 (12)	0.0356 (13)	0.0026 (10)	0.0014 (9)	−0.0067 (10)
C5	0.0429 (12)	0.0432 (12)	0.0279 (12)	0.0038 (10)	0.0026 (9)	−0.0006 (9)
C6	0.0386 (11)	0.0378 (12)	0.0288 (12)	0.0050 (9)	0.0026 (8)	−0.0015 (8)
C7	0.0427 (11)	0.0380 (11)	0.0258 (11)	0.0029 (10)	0.0032 (8)	−0.0015 (9)
C8	0.0392 (11)	0.0380 (12)	0.0364 (12)	0.0033 (9)	0.0063 (9)	−0.0007 (9)
C9	0.0515 (13)	0.0488 (14)	0.0396 (14)	−0.0017 (11)	0.0056 (10)	0.0038 (11)
C10	0.0593 (15)	0.0536 (15)	0.0581 (18)	−0.0024 (12)	0.0169 (13)	0.0125 (12)
C11	0.0476 (13)	0.0478 (14)	0.075 (2)	−0.0085 (11)	0.0117 (13)	0.0008 (13)
C12	0.0479 (14)	0.0542 (15)	0.0637 (18)	−0.0065 (12)	−0.0030 (12)	−0.0065 (13)
C13	0.0534 (14)	0.0517 (15)	0.0412 (14)	−0.0063 (12)	0.0002 (11)	0.0012 (11)

### Geometric parameters (Å, °)

**Table:**

O1—C1	1.350 (3)	C6—C7	1.498 (3)
O2—N1	1.229 (3)	C8—C13	1.390 (3)
O3—N1	1.221 (3)	C8—C9	1.386 (3)
O4—C7	1.243 (3)	C9—C10	1.387 (4)
O1—H1	0.8200	C10—C11	1.373 (4)
N1—C4	1.457 (3)	C11—C12	1.376 (4)
N2—C8	1.420 (3)	C12—C13	1.377 (3)
N2—C7	1.333 (3)	C2—H2	0.9300
N2—H2A	0.8600	C3—H3	0.9300
C1—C6	1.408 (3)	C5—H5	0.9300
C1—C2	1.393 (3)	C9—H9	0.9300
C2—C3	1.367 (3)	C10—H10	0.9300
C3—C4	1.389 (3)	C11—H11	0.9300
C4—C5	1.369 (3)	C12—H12	0.9300
C5—C6	1.391 (3)	C13—H13	0.9300
			
O1···N2	2.675 (2)	C8···C11^viii^	3.480 (3)
O1···O4^i^	2.609 (2)	C8···C6^vii^	3.583 (3)
O2···C11^ii^	3.335 (4)	C8···C1^vii^	3.557 (3)
O3···C3^iii^	3.364 (3)	C9···C7^vii^	3.339 (3)
O4···C2^iv^	3.212 (3)	C9···O4	2.853 (3)
O4···C9	2.853 (3)	C9···C6^vii^	3.556 (3)
O4···O1^iv^	2.609 (2)	C10···C7^vii^	3.501 (3)
O4···C1^iv^	3.319 (3)	C11···C8^vii^	3.480 (3)
O1···H2A	1.9500	C11···C3^xi^	3.599 (4)
O2···H3	2.4500	C11···O2^xii^	3.335 (4)
O2···H12^v^	2.7300	C1···H2A	2.5600
O2···H11^ii^	2.5900	C7···H9	2.8100
O3···H5	2.4000	C7···H1^iv^	2.7800
O3···H12^vi^	2.7800	H1···H2	2.2400
O3···H2^iii^	2.8300	H1···O4^i^	1.7900
O3···H3^iii^	2.7300	H1···C7^i^	2.7800
O4···H5	2.3900	H1···H5^i^	2.4800
O4···H9	2.2600	H2···H1	2.2400
O4···H1^iv^	1.7900	H2···O3^x^	2.8300
O4···H2^iv^	2.5400	H2···O4^i^	2.5400
N2···O1	2.675 (2)	H2A···O1	1.9500
N2···C1^vii^	3.426 (3)	H2A···C1	2.5600
C1···N2^viii^	3.426 (3)	H2A···H13	2.2600
C1···C8^viii^	3.557 (3)	H3···O2	2.4500
C1···O4^i^	3.319 (3)	H3···O3^x^	2.7300
C2···O4^i^	3.212 (3)	H5···O3	2.4000
C3···C6^viii^	3.478 (3)	H5···O4	2.3900
C3···C11^ix^	3.599 (4)	H5···H1^iv^	2.4800
C3···O3^x^	3.364 (3)	H9···O4	2.2600
C6···C8^viii^	3.583 (3)	H9···C7	2.8100
C6···C3^vii^	3.478 (3)	H11···O2^xii^	2.5900
C6···C9^viii^	3.556 (3)	H12···O2^xiii^	2.7300
C7···C10^viii^	3.501 (3)	H12···O3^xiv^	2.7800
C7···C9^viii^	3.339 (3)	H13···H2A	2.2600
			
C1—O1—H1	109.00	N2—C8—C13	116.1 (2)
O2—N1—O3	123.5 (2)	C9—C8—C13	119.5 (2)
O2—N1—C4	117.9 (2)	C8—C9—C10	119.2 (2)
O3—N1—C4	118.6 (2)	C9—C10—C11	121.2 (2)
C7—N2—C8	128.92 (18)	C10—C11—C12	119.5 (2)
C8—N2—H2A	116.00	C11—C12—C13	120.3 (2)
C7—N2—H2A	116.00	C8—C13—C12	120.3 (2)
O1—C1—C2	120.8 (2)	C1—C2—H2	119.00
O1—C1—C6	119.16 (19)	C3—C2—H2	119.00
C2—C1—C6	120.04 (19)	C2—C3—H3	121.00
C1—C2—C3	121.5 (2)	C4—C3—H3	121.00
C2—C3—C4	118.2 (2)	C4—C5—H5	120.00
N1—C4—C3	119.6 (2)	C6—C5—H5	119.00
N1—C4—C5	118.7 (2)	C8—C9—H9	120.00
C3—C4—C5	121.7 (2)	C10—C9—H9	120.00
C4—C5—C6	120.9 (2)	C9—C10—H10	119.00
C5—C6—C7	116.10 (18)	C11—C10—H10	119.00
C1—C6—C7	126.17 (19)	C10—C11—H11	120.00
C1—C6—C5	117.73 (19)	C12—C11—H11	120.00
O4—C7—N2	122.28 (19)	C11—C12—H12	120.00
O4—C7—C6	119.55 (18)	C13—C12—H12	120.00
N2—C7—C6	118.16 (18)	C8—C13—H13	120.00
N2—C8—C9	124.4 (2)	C12—C13—H13	120.00
			
O3—N1—C4—C3	−173.4 (2)	N1—C4—C5—C6	178.9 (2)
O2—N1—C4—C5	−171.8 (2)	C3—C4—C5—C6	−0.4 (3)
O2—N1—C4—C3	7.4 (3)	C4—C5—C6—C1	0.8 (3)
O3—N1—C4—C5	7.4 (3)	C4—C5—C6—C7	−178.9 (2)
C8—N2—C7—C6	179.93 (17)	C1—C6—C7—N2	4.2 (3)
C7—N2—C8—C9	−6.5 (3)	C5—C6—C7—O4	2.4 (3)
C8—N2—C7—O4	1.4 (3)	C5—C6—C7—N2	−176.16 (19)
C7—N2—C8—C13	174.8 (2)	C1—C6—C7—O4	−177.2 (2)
C6—C1—C2—C3	0.8 (3)	N2—C8—C9—C10	−178.7 (2)
O1—C1—C6—C5	179.3 (2)	C13—C8—C9—C10	0.0 (4)
O1—C1—C2—C3	−179.5 (2)	N2—C8—C13—C12	179.3 (2)
C2—C1—C6—C5	−1.0 (3)	C9—C8—C13—C12	0.5 (4)
C2—C1—C6—C7	178.6 (2)	C8—C9—C10—C11	0.1 (4)
O1—C1—C6—C7	−1.2 (3)	C9—C10—C11—C12	−0.7 (4)
C1—C2—C3—C4	−0.3 (3)	C10—C11—C12—C13	1.1 (4)
C2—C3—C4—C5	0.1 (3)	C11—C12—C13—C8	−1.0 (4)
C2—C3—C4—N1	−179.1 (2)		

Symmetry codes: (i) *x*, −*y*+2, *z*−1/2; (ii) *x*+1, *y*+2, *z*; (iii) *x*, −*y*+3, *z*+1/2; (iv) *x*, −*y*+2, *z*+1/2; (v) *x*+1, −*y*+2, *z*+1/2; (vi) *x*+1, −*y*+1, *z*+1/2; (vii) *x*, *y*−1, *z*; (viii) *x*, *y*+1, *z*; (ix) *x*+1, *y*+1, *z*; (x) *x*, −*y*+3, *z*−1/2; (xi) *x*−1, *y*−1, *z*; (xii) *x*−1, *y*−2, *z*; (xiii) *x*−1, −*y*+2, *z*−1/2; (xiv) *x*−1, −*y*+1, *z*−1/2.

### Hydrogen-bond geometry (Å, °)

**Table:**

*D*—H···*A*	*D*—H	H···*A*	*D*···*A*	*D*—H···*A*
O1—H1···O4^i^	0.82	1.79	2.609 (2)	176
N2—H2A···O1	0.86	1.95	2.675 (2)	141
C2—H2···O4^i^	0.93	2.54	3.212 (3)	130
C5—H5···O4	0.93	2.39	2.729 (3)	101
C9—H9···O4	0.93	2.26	2.853 (3)	121
C11—H11···O2^xii^	0.93	2.59	3.335 (4)	137

Symmetry codes: (i) *x*, −*y*+2, *z*−1/2; (xii) *x*−1, *y*−2, *z*.
